# Data Resource Profile: The Norwegian Armed Forces Health Registry (NAFHR)

**DOI:** 10.1093/ije/dyag132

**Published:** 2026-08-02

**Authors:** Hye Jung Choi, Matthieu Clauss, Elin Anita Fadum, Leif Aage Strand, Siri Eldevik Håberg, Kristine Vejrup

**Affiliations:** Norwegian Armed Forces Joint Medical Services, Military Medicine and Development Section, Sessvollmoen, Norway; Department of Community Medicine and Global Health, University of Oslo, Oslo, Norway; Norwegian Armed Forces Joint Medical Services, Military Medicine and Development Section, Sessvollmoen, Norway; Norwegian Armed Forces Joint Medical Services, Military Medicine and Development Section, Sessvollmoen, Norway; Norwegian Armed Forces Joint Medical Services, Military Medicine and Development Section, Sessvollmoen, Norway; The Centre for Fertility and Health, The Norwegian Institute of Public Health, Oslo, Norway; Department of Global Public Health and Primary Care, University of Bergen, Bergen, Norway; Norwegian Armed Forces Joint Medical Services, Military Medicine and Development Section, Sessvollmoen, Norway

**Keywords:** Norwegian Armed Forces Health Registry, NAFHR, early adulthood, conscripts, Norway

Key Features

- The Norwegian Armed Forces Health Registry (NAFHR) contains standardized health and performance assessment data for a large proportion of Norwegians in early adulthood (ages 17–19 years).
- It is updated regularly and linked annually, via the national personal identification number, to mortality (Cause of Death Registry) and migration (Population Register); linkage to other national registries is also possible.
- By incorporating existing military databases and digitized historical conscription records, NAFHR includes those who attend conscription board examinations, corresponding to >90% of men born in Norway (1946–91); few women from these cohorts are included because women’s participation was voluntary. After self-declaration-based preselection and gender-neutral conscription, the proportion is ∼40% for men and ∼30% for women born from 1992 onwards.
- All Norwegians born from 1992 onwards are required to submit an electronic self-declaration form at age 17 years. Responses cover ∼100% of each birth cohort: 83%–98% submit at age 17 years, with the remainder completing it within 1–2 years, effectively serving as a population-based survey.
- NAFHR contains individual-level data from conscription board examinations (physical and cognitive profiles and 10 health domains) and from electronic self-declaration (self-reported health, lifestyle, and social factors).
- Data are available upon application; ethics approval(s) may be required.

## Data resource basics

### Background

The purpose of the Norwegian Armed Forces Health Registry (NAFHR) is to monitor the health and health trends of military personnel but its scope extends beyond service members. It includes mandatory self-declaration at age 17 years, national conscription board examinations, and service records, providing broad coverage and detailed health information for occupational epidemiology and population-based research. NAFHR was established in 2005 to characterize service-related health risks and to provide a robust data source for research and statistics on Armed Forces personnel health [1, 2]. As a national health register, NAFHR collects and processes information without individual consent in accordance with the Norwegian Health Register Act [3]. The Norwegian Ministry of Defence serves as the formal data controller, while the Norwegian Armed Forces Medical Services acts as the data processor [2].

### Conscription procedures and birth-cohort coverage over time in NAFHR

**Figure 1:**
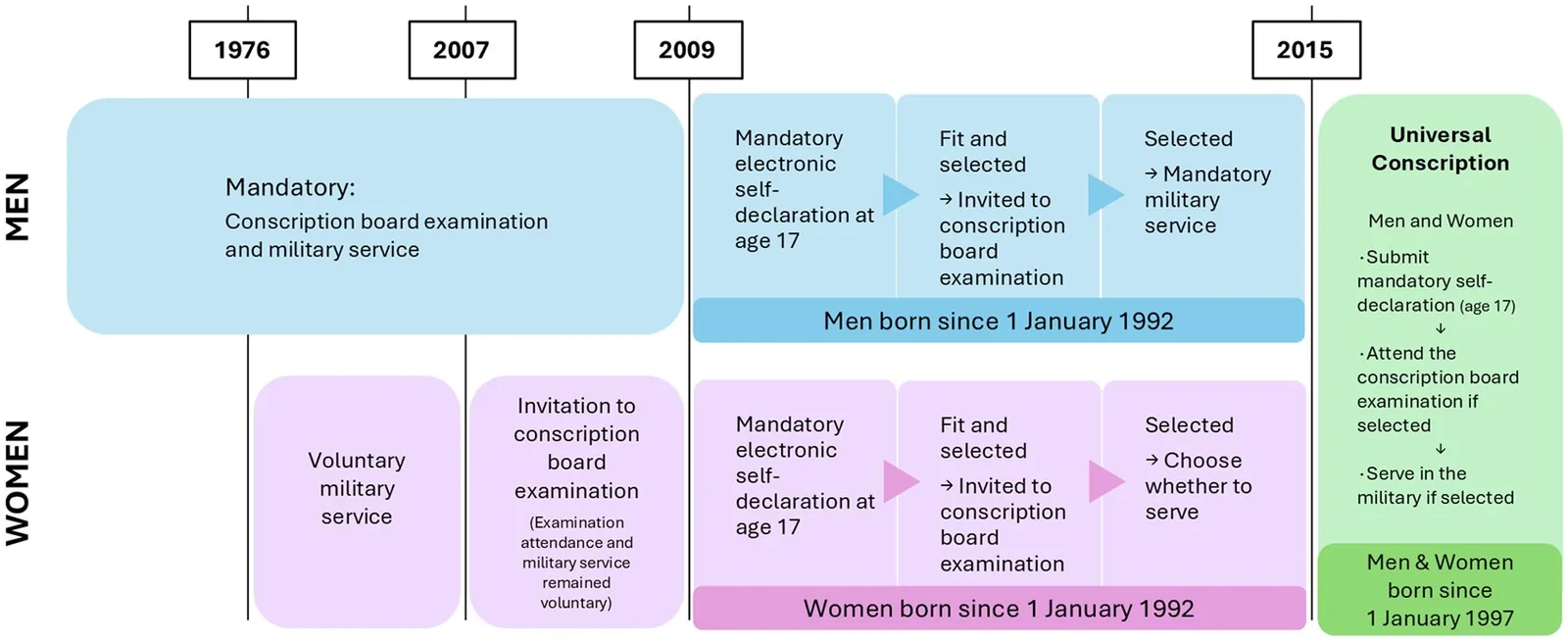
Changes in Norwegian conscription legislation and policy over time.

The composition of conscripts recorded in NAFHR has evolved over time in response to changes in conscription laws and procedures [4], with each change and its impact on the affected cohorts illustrated (Fig. 1). NAFHR data on Norwegians in early adulthood are derived from two sources: in-person conscription board examinations and a mandatory electronic self-declaration covering health, lifestyle, and motivation, according to the Norwegian Defence Act [5] and the Conscription Regulations [6].

NAFHR includes health information on all Norwegians who have undergone physical conscription board examinations prior to mandatory military service, conferring extensive population coverage [1]. While the standard age for physical conscription examinations is 17–19 years, some individuals are postponed for education or health reasons. Immigrants to Norway who are still within the eligible age range for military service may also undergo the board examination at older ages, although such cases are rare. As of February 2025, 1.82 million conscripts had been registered in NAFHR, all of whom had personal identification numbers that enabled linkage within NAFHR to military service and international operation records, as well as to external national registries and databases, including the Norwegian Cause of Death Registry, Cancer Registry, the Patient Registry, and databases maintained by Statistics Norway (SSB).

NAFHR also includes responses from a mandatory electronic self-declaration, which has been collected from Norwegians born since 1 January 1992 [4] (Fig. 1). The form is used for the initial screening of individuals’ suitability for military service and to determine who will be invited to attend the conscription board examinations. Accordingly, all 17-year-olds receive the self-declaration and the respondents confirm that they understand that providing incorrect or misleading information may result in criminal liability under the Norwegian Defence Act [5]. Before this system was introduced, nearly all men underwent conscription board examinations, with exemptions mainly for severe disability or criminal history. Under the current system, the self-declaration form is sent to ∼60 000 individuals each year and ∼24 000–25 000 are invited to the conscription examination, depending on the Armed Forces’ needs and requirements.

#### Pre-1950 birth cohorts

**Figure 2:**
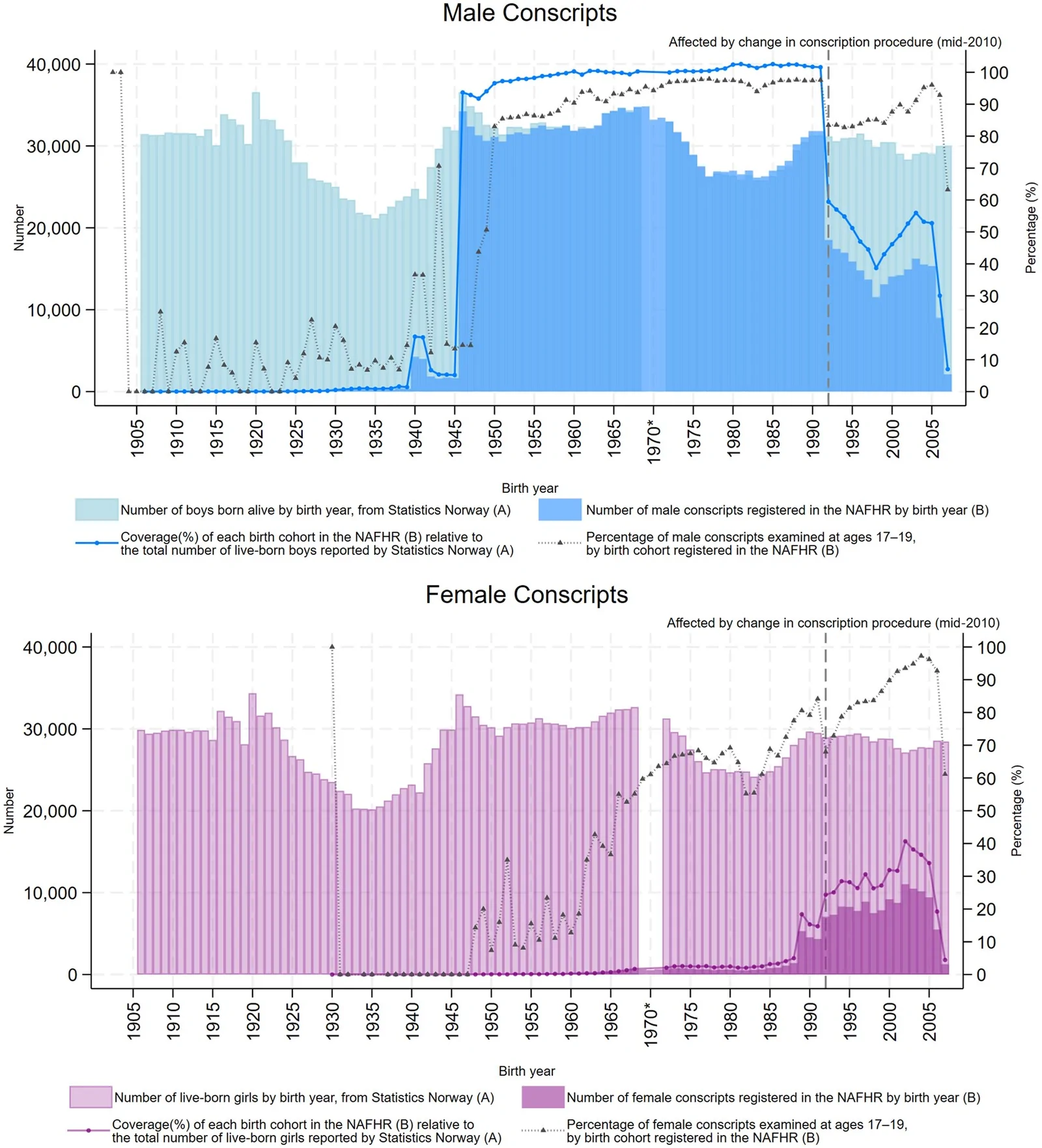
Number of male (top) and female (bottom) conscripts attending conscription board health examinations in Norway by birth year in NAFHR. Footnote: *Data on live-born boys and girls for 1969–71 are not available from Statistics Norway (SSB).

The first conscripts recorded in NAFHR were born in 1902. However, only small numbers are recorded through the 1945 birth cohort. Beginning with the 1946 birth cohort, NAFHR provides broadly representative coverage of the Norwegian male population (Fig. 2), although very limited information on physical profiles is recorded (Table 1). The recorded examination year appears to be inaccurate for many individuals, making it impossible to determine the examinee’s age at evaluation and resulting in a low proportion of conscripts aged 17–19 years in cohorts born before 1950.

**Table 1:** Number (%) of conscripts with available data from conscription board health examination conducted at ages 17–19^a^ years in NAFHR.

Birth cohorts	Total conscripts registered in NAFHR [A]	Conscripts with available data measured at ages 17–19 years [B] among total registered conscripts [A]								
		Conscripts aged 17–19 years [B]	Sex	Height	Weight	General ability score	Swim	Ski	Aerobic fitness	Isometric muscle strength
1902–39	2708	277 (10.2)	277 (10.2)	20 (0.7)	4 (0.1)	8 (0.3)	6 (0.2)	5 (0.2)	4 (0.1)	–
1940–49	143 673	43 725 (30.4)	43 725 (30.4)	8440 (5.9)	156 (0.1)	6780 (4.7)	7756 (5.4)	7084 (4.9)	428 (0.3)	–
1950–59	317 825	274 971 (86.5)	274 971 (86.5)	234 846 (73.9)	212 293 (66.8)	215 000 (67.6)	227 987 (71.7)	207 583 (65.3)	210 137 (66.1)	–
1960–69	338 065	313 531 (92.7)	313 531 (92.7)	309 102 (91.4)	308 922 (91.4)	301 223 (89.1)	302 156 (89.4)	301 894 (89.3)	302 568 (89.5)	–
1970–79	304 741	293 047 (96.2)	293 047 (96.2)	285 288 (93.6)	284 966 (93.5)	280 719 (92.1)	281 852 (92.5)	281 140 (92.3)	283 007 (92.9)	–
1980–89	289 206	276 911 (95.7)	276 911 (95.7)	267 928 (92.6)	267 710 (92.6)	258 556 (89.4)	267 128 (92.4)	266 534 (92.2)	264 466 (91.4)	5 (0)
1990–99	257 403	221 715 (86.1)	221 715 (86.1)	216 636 (84.2)	216 713 (84.2)	207 724 (80.7)	216 643 (84.2)	212 517 (82.6)	193 031 (75)	11 833 (4.6)
2000–07^b^	167 191	153 582 (91.9)	153 582 (91.9)	140 400 (84)	140 399 (84)	138 750 (83)	140 526 (84.1)	131 535 (78.7)	138 105 (82.6)	138 520 (82.9)

a The numbers and percentages represent the completeness among conscripts who attended the board examination at ages 17–19 years, following legislative criteria, within the corresponding birth cohorts registered in NAFHR. Consequently, these figures would increase—approaching the total registered number [A]—if they included conscripts examined after age 19 years due to education- or work-related deferments.

b By February 2025, not all individuals born in 2006–7 had been summoned for conscription examinations and therefore were not yet included in NAFHR.

#### 1950–91 birth cohorts

Around 95% of all men born in Norway in 1950–91 are registered in NAFHR and ∼80%–90% of male conscripts were examined at ages 17–19 years, indicating that the data largely reflect early adulthood (Fig. 2). Data include various measures collected at the conscription examination site, including physical profiles, cognitive ability, and health-profile domains (Tables 1 and 2). Women were allowed to serve voluntarily in noncombat roles in 1976 [7] and all military positions were opened in 1985 [4]. Since then, few women have volunteered for conscription and service (Fig. 2).

**Table 2:** Number (%) of conscripts with available data in 10 health-profile items from conscription board health examination conducted at ages 17–19^a^ years in NAFHR.

Birth cohorts	Total conscripts registered in NAFHR [A]	Conscripts with available data measured at ages 17–19 [B] among total registered conscripts [A]										
		Conscripts aged 17–19 years [B]	General health	Digestion	Eye/vision	Ear/hearing	Arm	Hand	Walking	Back	Skin	Mental health
1902–39	2708	277 (10.2)	8 (0.3)	8 (0.3)	8 (0.3)	8 (0.3)	8 (0.3)	8 (0.3)	8 (0.3)	8 (0.3)	8 (0.3)	8 (0.3)
1940–49	143 673	43 725 (30.4)	113 (0.1)	111 (0.1)	111 (0.1)	111 (0.1)	111 (0.1)	111 (0.1)	112 (0.1)	112 (0.1)	111 (0.1)	111 (0.1)
1950–59	317 825	274 971 (86.5)	61 (0)	60 (0)	60 (0)	60 (0)	60 (0)	60 (0)	60 (0)	60 (0)	60 (0)	62 (0)
1960–69	338 065	313 531 (92.7)	275 166 (81.4)	274 891 (81.3)	274 903 (81.3)	274 898 (81.3)	274 875 (81.3)	274 875 (81.3)	274 902 (81.3)	274 912 (81.3)	274 879 (81.3)	275 425 (81.5)
1970–79	304 741	293 047 (96.2)	288 692 (94.7)	287 996 (94.5)	288 040 (94.5)	288 032 (94.5)	287 967 (94.5)	287 970 (94.5)	288 028 (94.5)	288 014 (94.5)	287 966 (94.5)	288 537 (94.7)
1980–89	289 206	276 911 (95.7)	274 724 (95)	274 559 (94.9)	274 569 (94.9)	274 552 (94.9)	274 550 (94.9)	274 546 (94.9)	274 562 (94.9)	274 562 (94.9)	274 555 (94.9)	274 946 (95.1)
1990–99	257 403	221 715 (86.1)	216 619 (84.2)	216 274 (84)	216 275 (84)	216 264 (84)	216 255 (84)	216 244 (84)	216 297 (84)	216 278 (84)	216 252 (84)	216 884 (84.3)
2000–07^b^	167 191	153 582 (91.9)	139 534 (83.5)	139 518 (83.4)	139 520 (83.4)	139 516 (83.4)	139 519 (83.4)	139 517 (83.4)	139 521 (83.5)	139 518 (83.4)	139 517 (83.4)	139 539 (83.5)

a The numbers and percentages represent the completeness among conscripts who attended the board examination at ages 17–19 years, following legislative criteria, within the corresponding birth cohorts registered in NAFHR. Consequently, these figures would increase—approaching the total registered number [A]—if they included conscripts examined after age 19 years due to education- or work-related deferments.

b By February 2025, not all individuals born in 2006–7 had been summoned for conscription examinations and therefore were not yet included in NAFHR.

#### 1992–96 birth cohorts

Men and women born on or after 1 January 1992 are subject to the mandatory self-declaration. While men and women were required to attend the physical conscription examination if invited based on their responses, women were still able to choose not to serve even if selected [4]. Consequently, from mid-2010 onwards, the number of men attending the board examination was roughly halved, while that of women attending the examination started to increase (Fig. 2). Despite the reduced number of individuals attending conscription examinations, the mandatory self-report policy ensures that the registry captures information at the birth-cohort level by including all individuals who submit the self-declaration form. According to SSB, ∼60 000 individuals have been born in Norway each year since 1992 [8] and the total number of individuals who submitted the self-declaration in each birth cohort is nearly comparable to the SSB figures (Supplementary Fig. S1).

#### 1997–present birth cohorts

In 2015, Norway became the first NATO country to introduce gender-neutral conscription [9] and those born since 1 January 1997 are subject to this policy. Women, like men, are now required to attend the conscription examination and serve if the Armed Forces requires it [4]. Therefore, the number of women attending the examination has increased, reaching 40% of women born in Norway in the corresponding birth year (Fig. 2). The sharp declines in the 2006–7 cohorts reflect that not all individuals had yet been summoned to conscription examinations as of February 2025, when the data were extracted for this review (Fig. 2).

### Follow-up

NAFHR is updated quarterly for conscription board examinations and monthly for self-declaration from military databases, whereas mortality (Norwegian Cause of Death Registry) and migration (Norwegian Population Register) data are incorporated annually. Individuals who enter military service following the conscription examination and are subsequently employed by the Norwegian Armed Forces can be followed longitudinally in NAFHR, enabling occupational and military epidemiological studies.

### Ethical clearance

The legal basis for NAFHR to store and manage registry data is set out in the Act of 18 May 2001 No. 24 on Personal Health Data Filing Systems and the Processing of Personal Health Data [3]. Researchers seeking to use data for medical and health research from NAFHR must obtain approval from the Regional Committees for Medical and Health Research Ethics before submitting an application to NAFHR.

## Data collected

### Data collected from the self-declaration form

The form typically includes 50–60 questions covering health, social skills, schooling, hobbies, language, and interest in military service. Since its introduction in 2009, the thematic structure has remained largely consistent, although the specific questions within each section have changed between years. Table 3 presents the questions that remained consistent for ≥10 years across the 2009-to-2023 form versions, by birth cohort with available information (Table 3). Although the standard requirement is submission at age 17 years and 83%–98% of respondents do so at that age, some submit it later, due to health, an apprenticeship, or immigration [10]. We present in Table 3 the number of 17-year-old submitters to follow the standard requirement; however, if submissions at older ages were included, then the total number of submitters would more closely match the number born in Norway 17 years ago, as shown in Supplementary Fig. S1.

**Table 3:** Number of 17-year-olds submitting the self-declaration form by birth cohort and form version with data availability (gray indicates available; white indicates not availabe).

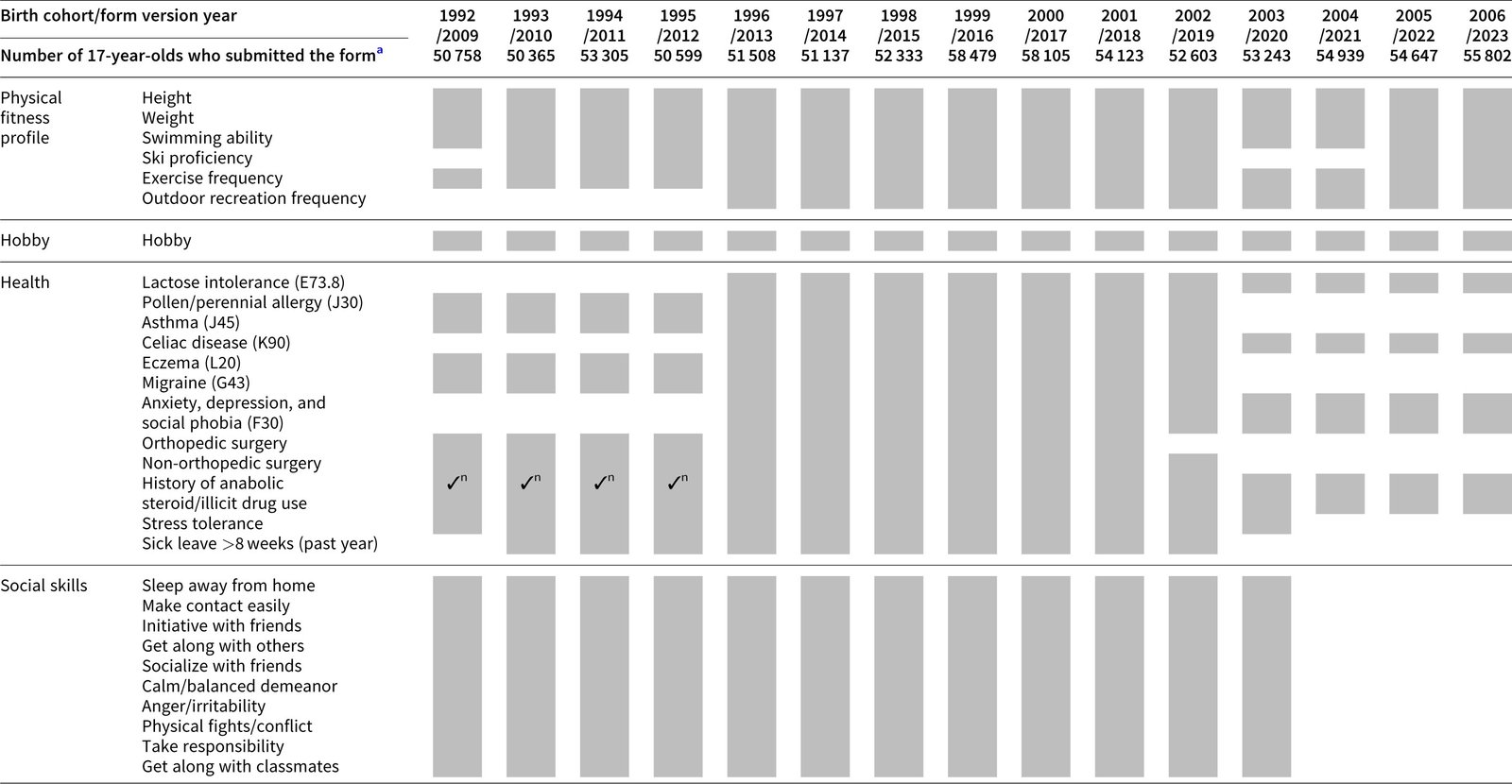

(ICD-10 codes); ✓^n^ is for illicit drug use only.

a These numbers include only individuals who submitted the form at age 17 years in each cohort, which is used for conscription evaluation in accordance with legal requirements; totals would be higher (∼60 000, comparable to the corresponding birth cohort each year) if submissions after age 17 years due to education or health reasons were included. However, as the questionnaire content has changed over time, we restrict the denominator to submissions from those aged 17 years, to maintain consistency across cohorts and form versions.

### Data collected from the conscription board examination

Information on conscripts and their health is collected during the conscription board examination, which is conducted using standardized procedures by trained military officers and physicians [4]. Historically, the conscription examinations were conducted locally in municipalities, with military medical teams traveling between sites with the necessary equipment. Since 2010, the examinations have been centralized at 10 regional military facilities [11].

The conscription board examination includes physical and cognitive assessments conducted according to standardized instructions from the Armed Forces Joint Medical Services [12]. As a result, the assessment items and measurement have remained largely unchanged over time and more detailed descriptions of each assessment are provided elsewhere [4, 12, 13]. Physical performance is tested and assessed by military officers (Table 1). Swimming ability (200 meters) and ski proficiency are self-reported, whereas aerobic fitness and isometric muscular strength are measured on-site during the examination. Aerobic fitness and strength are primarily recorded on a stanine scale ranging from 1 (worst) to 9 (best). When regular testing is not feasible, a three-level scale (A–C), reflecting an overall and more subjective evaluation, can be used [4, 14]. Aerobic fitness testing methods have changed over time, using a bicycle ergometer test (1969–85), a treadmill test (1988–93), periods in which aerobic fitness was assessed based on athletic background and physical constitution, and a treadmill since 2011 [4]. Isometric muscle strength testing was introduced in 2011 [4]; therefore, test results are available primarily for birth cohorts from 1994 onward.

A measure of cognitive ability, often referred to as a “general ability test score” or IQ, is a standardized composite score derived from three time-limited subtests assessing skills in arithmetic (25 min), figure problem solving (20 min), and word similarities (8 min) [4]. A stanine score, consistent with the scale used for aerobic fitness and muscular strength tests, is applied. When direct testing is not feasible (e.g. due to dyslexia), an A–C scale based on an overall assessment of the conscript’s background and results from the figure subscale is used [12].

A health profile is generated based on the assessment of 10 organ-specific body systems, defined by the Armed Forces Joint Medical Services as general physical health, vision, hearing, musculoskeletal function, skin, and psychological health [4]. The evaluation is conducted by a physician in the examination room (Table 2). Based on the physical assessment, individuals are classified in 10 body systems as having normal, insignificant, minor-to-moderate, or major impairment, or as unfit for military service [4]. Since the 1970s, medical conditions causing functional impairment have been recorded by using the International Classification of Diseases (ICD) by military physicians [4].

### Registered in NAFHR

NAFHR incorporated data from existing Armed Forces military databases and digitized historical records, enabling the inclusion of records predating its establishment [11, 15, 16]. Thereafter, individual conscript records from the self-declaration form and conscription examination were submitted to the Norwegian Armed Forces Personnel and Conscription Centre and subsequently transferred to NAFHR. NAFHR staff conduct data-quality checks to identify inconsistencies and ensure periodic updates with the Norwegian Cause of Death Registry (mortality) and the Norwegian Population Register (migration).

As part of this study, we conducted quality checks of NAFHR data. The variables presented here showed good completeness and consistent use of categorical scales over time among registered conscripts, supporting their suitability for research. A small number of extreme values were observed for self-reported height and weight in the electronic self-declaration, likely reflecting respondent entry errors.

## Data resource use

With broad population coverage of Norwegian and unique information capturing characteristics in early adulthood, NAFHR data have been extensively used in internal military quality-control studies for veterans as well as a range of epidemiological studies. They have been used to estimate prevalence, examine trends, and identify factors associated with adolescent health conditions for which national-level data sources are lacking—such as mental distress, self-harm, overweight, or health problems for which individuals rarely seek medical care [17–19].

By linking NAFHR to national registries and databases, researchers have examined associations between early adulthood health and later-life outcomes, including suicide [20] and early receipt of social benefits [21]. Linkage to family information from SSB has also allowed examination of how family factors are associated with conscripts’ health [22] and health outcomes among their extended family members [23].

Furthermore, women constitute an increasing proportion of the Armed Forces, as reflected by the adoption of mandatory gender-neutral conscription across all Scandinavian countries [24]. Yet, military women remain understudied, often due to limited statistical power or a lack of reliable data sources [25]. With gender-neutral conscription, the growing number of women provides a new opportunity for longitudinal and high-quality cohort studies of women’s health in military and public health contexts.

## Strengths and weaknesses

NAFHR is a unique and valuable resource for studying the health and physical profiles of Norwegians in early adulthood, drawing on data collected through mandatory military service and the legally required self-declaration form at age 17 years. Information from both the self-declaration form and the conscription examination can help elucidate how early-life factors relate to later-life outcomes through linkage with other registries and databases, and can be used to examine trends across birth cohorts. This is facilitated by NAFHR’s broad population coverage and standardized timing and measurements over time.

In contrast to morbidity registries, which only capture individuals who develop disease, NAFHR provides basic and early health and physical assessments for a large share of the general population through the conscription system. In particular, mental health measures and general ability scores have received considerable attention. Furthermore, health-profile measurements are used not only during the conscription board examination, but also throughout military service; therefore, they provide a useful indicator of changes in individual health statuses over time and across generations.

NAFHR also has strong potential for international research collaboration because Sweden [26] and Denmark [27] maintain comparable conscription registers with similar measures collected at similar ages. This facilitates cross-country comparisons and pooled analyses to assess the robustness and generalizability of findings across Nordic populations.

However, there are several limitations. Not all birth cohorts are equally well represented in NAFHR. For cohorts born before 1950, only a small portion is included and their recorded health information is sparse. For cohorts born in 1992 or later, the call to the conscription board examination is selective and based upon information from the self-declaration form; it is thus restricted to individuals considered healthy and suitable for military service. As a result, for research on women and men born before 1950 or after 1991, those who underwent the conscription examination are not representative of the general population.

Still, data from the electronic form may serve as an alternative to conscription examination data, although the items are self-reported. All Norwegians are legally required to submit the self-declaration form at age 17 years; data have national coverage and very high response rates that are similar for both sexes. Consequently, the data are largely free from selection bias or low response rates, which are common limitations of many surveys. This highlights the value of the self-reported data as a population-based survey resource.

Despite routine quality checks and periodic updates, documentation is limited on how conscripts’ records predating NAFHR’s establishment in 2005 were digitized and entered into the registry. To improve data completeness, conscription examination records for ∼17 000 Norwegian men born in 1950 were digitized; the procedures are well documented and available [11]. However, records for individuals born before 1950 were affected by inaccurate dates, resulting in very few conscripts appearing eligible at ages 17–19 years. This limitation should therefore be considered in studies that require precise age information from NAFHR, particularly for those cohorts born before 1950.

## Data resource access

Applicants must submit an application form and a project description, along with copies of all relevant approvals/permits and the corresponding applications. International researchers without a Norwegian collaborating institution should consult NAFHR to clarify applicable data-access procedures. Further information is available on the NAFHR website (https://www.forsvaret.no/forskning/forsvarets-helseregister-ime). Questions regarding data access and the application may be directed to fsan.fhr.datautlevering@mil.no, or Kristine Vejrup (leader, kvejrup@mil.no).

## Ethics approval

This investigation was conducted as a quality-control project and therefore did not require approval from the Regional Committees for Medical and Health Research Ethics. The work was authorized under the NAFHR regulations and carried out by NAFHR employees, who are authorized to process the data without the data subjects’ consent, in accordance with the Health Register Act, applicable regulations, and the NAFHR internal procedure for secure data management.

## Supplementary Material

dyag132_Supplementary_Data

## Data Availability

The data are not publicly available due to legal and ethical restrictions. Access may be granted upon reasonable request, subject to approval through a formal application process to the data holder and any required ethics approvals. Further information on accessing NAFHR data, including the application procedure, is available from the Norwegian Armed Forces Health Register (https://www.forsvaret.no/forskning/forsvarets-helseregister-ime/forskning-og-data).
