# Supplementary material for: Data Resource Profile: The Norwegian Armed Forces Health Registry (NAFHR)
Source: Int J Epidemiol. 2026 Aug 2;55(4):dyag132. doi: 10.1093/ije/dyag132 (PMC13428907; doi:10.1093/ije/dyag132)

Supplementary Figure S1. Number of individuals born in Norway since 1992, those subject to the mandatory self-declaration policy for conscripts, and the total number who submitted the declaration by birth year


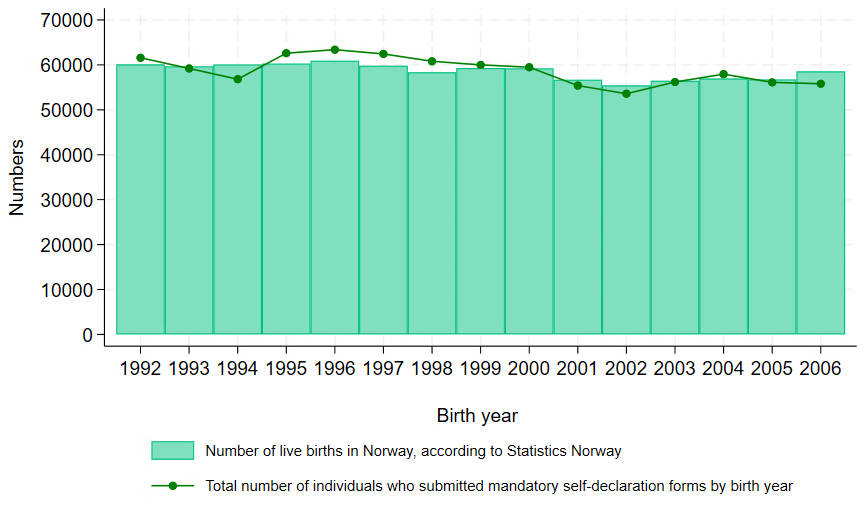

Supplement: dyag132_Supplementary_Data [file dyag132_supplementary_data.docx]
